# Their wellbeing affects our wellbeing: student perspectives of lecturer wellbeing and its consequences for student wellbeing

**DOI:** 10.1007/s10734-024-01365-0

**Published:** 2024-12-14

**Authors:** Katie E. Rakow, Michael Priestley, Nicola C. Byrom, Juliet L. H. Foster, Eleanor J. Dommett

**Affiliations:** 1 Department of Psychology, Institute of Psychiatry, Psychology and Neuroscience, King’s College London, London, SE1 1UL UK; 2 Centre for Technology Enhanced Learning, King’s College London, London, SE1 9NH UK; 3 School of Education, Durham University, Durham, UK

**Keywords:** University culture, Academics, Faculty, Undergraduates, Wellbeing, Mental health

## Abstract

A “whole university approach” has been recommended for addressing concerns about the wellbeing of UK university lecturers and students. Previously, staff wellbeing has been explored from staff perspectives. Student wellbeing has been explored from the perspectives of both students and staff. However, little research has been conducted on student perspectives on staff wellbeing and its possible impact on students. Addressing this gap, this study explored student perceptions of their lecturers’ wellbeing and ways that it can impact on student wellbeing. Three themes were identified from the thematic analysis of 9 focus groups with 41 undergraduate students. First, students notice their lecturer wellbeing, particularly if they have the opportunity to interact with their lecturers. Second, students perceive that their lecturers’ wellbeing can be affected by university policies and practices, student behaviours, and external factors. Third, lecturer wellbeing and student wellbeing are often reciprocal, emphasising the importance of lecturer-student interactions. These findings have implications for a whole university approach, namely development of university practices that support sufficient time for constructive lecturer-student interactions, such as timetabling, allocation of administrative tasks, class size and staffing levels.

**Supplementary Information:**

The online version contains supplementary material available at 10.1007/s10734-024-01365-0.

## Introduction

Research findings continue to highlight concerns about university academics’ wellbeing. Cross-sectional surveys conducted in Australia and the UK have reported academics experiencing wellbeing levels below the national average (Fetherston et al., 2020; Wray & Kinman, 2021). Similarly, trends over the past decade indicate rising levels of poor mental health and lower wellbeing among UK university students (Lewis & Bolton, 2023; Tabor et al., 2021). These trajectories for academics have persisted worldwide post-Covid 19 (Hammoudi Halat et al., 2023).

Wellbeing is a complex and contested concept. Definitions emphasise that good wellbeing transcends solely the absence of mental distress (Huppert, 2009). For this project, wellbeing was defined as a multifaceted construct comprised of hedonia and eudaimonia, often summarised as “feeling good and functioning well” (Ruggeri et al., 2020). While hedonic components include emotions, mood, and satisfaction with life, eudaimonia includes sense of competence, engagement and interest in activities, commitment to meaningful goals and activities, and supporting others and being supported (Diener et al., 2010; Dodd et al., 2021; Ng Fat et al., 2017).

Several studies have sought to explain the antecedents of lecturer and student wellbeing. Results from a systematic review, 2009–2019, of 28 quantitative and qualitative studies from multiple countries suggest that lecturers perceive workload to be their principal work-related stressor (Urbina-Garcia, 2020). Other surveys indicate that academics are at a higher risk of work overload and work-life conflict and report lower levels of job control and more work-related stress compared with non-academic staff (Fontinha et al., 2019; Johnson et al., 2019). Lecturer-student interactions (Hammoudi Halat et al., 2023) and student behaviour (Urbina-Garcia, 2020; Watts & Robertson, 2011) may contribute to lecturer wellbeing.

Focus groups with university students (Priestley et al., 2022), university staff (Brewster et al., 2021), and university students and staff (Jones et al., 2020) suggest that academic staff mental health and wellbeing impact on students’ learning, mental health, and wellbeing. Priestley et al. (2022) reported that students attributed academics’ lack of empathy or inconsistent marking to high workload and limited support. Brewster et al., (2021, p.3) found that university staff reported that university structures, policies, and culture contributed to their workload and poor wellbeing, concluding that staff wellbeing was “integral” to and “co-dependent” with student wellbeing.

Research suggests that teacher-student interactions are important for the wellbeing of academics *and* students (Kiltz et al., 2020). University students perceive that lecturers’ interactions with their students, as shown through their teaching practices, availability, approachability, empathy, and communication skills, contribute significantly to student wellbeing (Baik et al., 2019; Eloff et al., 2021). Findings from a longitudinal study with 3 cohorts of undergraduate students from 46 American colleges suggested that frequency *and* quality of teacher-student interactions predicted student wellbeing (Trolian et al., 2020). Lecturers perceive indicators of quality interactions to include approachability, mutual engagement, trust, and respect (Hagenauer et al., 2023).

Studies indicate an interdependence between teacher and student wellbeing (Harding et al., 2019). For example, evidence from a longitudinal study in 20 German secondary schools (Frenzel et al., 2018) supports the concept of emotion transmission, including the assumption that another’s emotions can be interpreted through their behaviour. In that study, when students perceived teacher enthusiasm, students subsequently reported enjoyment. Moreover, when teachers perceived student engagement, teachers subsequently reported enjoyment. Therefore, and consistent with our conceptualisation of wellbeing as “feeling good and functioning well” (Ruggeri et al., 2020), we invited students to interpret their lecturers’ wellbeing, including their emotions, based on their experiences of their lecturers’ behaviour.

In secondary schools, this interactive relationship between teacher and student wellbeing (Harding et al., 2019) has been suggested to both shape and be shaped by the school climate (Gray et al., 2017). The concept of a school climate captures the “norms, goals, values, interpersonal relationships, teaching and learning practices and organisational structures” of a school (Thapa et al., 2013, p. 358). In Higher Education, a “whole institution” approach has been recommended to change institutional climate and promote the wellbeing of the entire university community (Hughes & Spanner, 2019). This encourages institutions to adapt organisational structures to better support staff wellbeing, recognising the circular impact this has on student wellbeing and then the whole community (Hughes & Spanner, 2019).

While existing research has explored lecturer perspectives on the interaction between lecturer and student wellbeing (e.g. Brewster et al., 2021), there is limited understanding of student perspectives on staff lecturer wellbeing and the possible interaction between student–lecturer wellbeing. To advance this understanding, this study examined the research question: What are student perspectives on lecturer wellbeing, and how might lecturer wellbeing impact on student wellbeing?

## Method

In March 2023, 41 full-time undergraduate students participated in 9 focus groups, ranging from 3 to 8 participants per group. Focus groups allow for students to consider different perspectives and experiences within a shared context (Gaskell, 2000) whilst alleviating interviewer-interviewee power dynamics. Based on the principles of information power, considering a broad research question, not testing a specific theory (Malterud et al., 2016), a minimum of 35 participants were recruited (Terry et al., 2017). A target of at least eight focus groups was set, so first-year and upper-year students could be grouped separately to reduce student–student power dynamics.

Participants were from two research-intensive universities, located in England and Scotland. Participants were recruited via email circulars and webpages within two UK universities and a UK-wide charity. Undergraduate students were invited to discuss the question “How does lecturer wellbeing impact undergraduate students?” Research ethics approval was granted by an Institutional Research Ethics Panel. Each participant received a £15.00 e-voucher upon the study’s completion.

Participants were 18–28 years old (mode 20 years) and self-identified as either female (*N* = 33) or male (*N* = 8). Each year of undergraduate study was represented: first (*N* = 14), second (*N* = 13), third (*N* = 9), fourth (*N* = 3), and final year (*N* = 2). Participants followed 17 different categories of degree major (see Supplementary Materials, Table S1). Participants identified with one of eight different ethnicities (Table S2), the most common being Asian or Asian British (*N* = 22). Twelve participants reported having a disability (Table S3).

Focus groups were structured around three main questions: (1) To what extent do you consider lecturer wellbeing when you interact with your lecturers? (2) What factors do you think impact on lecturer wellbeing, positively or negatively? (3) Do you think lecturer wellbeing impacts on your experiences as a student? Prompts were used for follow-up discussion (Supplementary Materials, Topic Guide). Participants were not asked to share their own personal experience of mental health difficulties. Because the term “wellbeing” is complex and multifaceted, the term was not defined by the facilitator to allow participants to share their own interpretations of their experiences. Focus groups were conducted online, recorded audio-visually (ranging from 33 to 49 min, average 43 min), transcribed verbatim and anonymised by one researcher (KR).

### Data analysis

A critical realist orientation was adopted because the research question was focused on students’ experiences and their interpretations of those experiences within the bounds of the university social context (Braun & Clarke, 2006). Reflexive thematic analysis with an inductive approach was used to generate themes, “patterns of shared meaning” (Braun & Clarke, 2019, p. 593). Theme generation involved iterative phases, all of which were conducted with reference to the research question (Braun & Clarke, 2006). Data analysis and theme development consisted of data familiarisation, using handwritten notes made during the focus group, transcribing the focus groups verbatim (KR), and generating codes independently from transcripts (KR & MP) for a “richer, more nuanced” understanding of the data (Braun & Clarke, 2019, p. 594). Agreement on themes was arrived at through an iterative process, involving discussion with reference to codes and data (KR & MP), and re-reading transcripts (all authors). Paper and pen, NVivo 12, and Microsoft Excel were used to generate the themes discussed below (see Supplementary Materials for additional supporting quotations). Braun and Clarke’s (2023) 20-point checklist for reflexive thematic analyses was used.

### Positionality

Authors 1 and 2 have worked at UK universities in a range of professional services, research, and teaching roles. Authors 3–5 hold senior teaching and research academic posts and have conducted extensive educational research.

## Results

Focus groups explored student perspectives on lecturer wellbeing and how lecturer wellbeing might impact on student wellbeing. The three themes identified related to: (i) the role of human connection; (ii) a complex range of factors impacting lecturer wellbeing; and (iii) the interdependence of lecturer wellbeing and student wellbeing. Taken together, the data patterns indicate that through the relational character of the learning process, students perceive a complex range of factors to impact lecturer wellbeing wherein positive lecturer wellbeing can enrich their own learning and wellbeing.

### Theme 1: “Wait, these are actually people that are working”—Human connection

Most participants had considered the wellbeing of at least one lecturer. Human connection between lecturers and students facilitated students’ awareness of their lecturers’ wellbeing. For many, a personal interaction with a lecturer or a communication about a lecturer that humanised their role “prompted” students’ awareness of their lecturer’s wellbeing (P10). For several students, consideration of lecturer wellbeing was limited to those with whom they felt a particular connection: “I don’t know if I thought about the wellbeing of all my lecturers and teachers, but, um, definitely for the ones that I had, or I felt more close to” (P2); “The better you know someone and the more you see them, like the better kind of (…) awareness you have towards their wellbeing” (P40).

For some students, this “human connection” was best supported via in-person interactions rather than pre-recorded or online teaching because “watch[ing] the lectures online, it’s much harder to perceive their emotions or wellbeing” (P16):

*They just felt like not human because (…), I was just watching them through my screen. Um, and then the only time like that I was able to understand, “Wait, these are actually people that are working and that I'm seeing every day, that are coming from home, that have got families” was when I went in on campus*. (P3)

Being lectured by several academics for one module or large class sizes introduced barriers to building relationships that limited awareness of lecturer wellbeing: “every week it’s a different lecturer. And we don’t really have much opportunity to get to know each of the lecturers” (P26); “when I do talk to them, it’s not very often. Like, I’m in a lecture-hall with about 400 other people, so I don’t have the chance to interact with them personally” (P32). However, announcements informing students that “this lecturer can’t be here for personal circumstances” ignited considerations of lecturer wellbeing: “due to a family emergency my lecturer can’t conduct the test. And then, I think, we were all thinking like, ‘I wonder what he’s going through. We hope he’s okay’.” (P32).

Some students noticed their lecturers’ wellbeing because of changes in their lecturers’ behaviour, physical appearance, mood, enthusiasm for the subject, energy (e.g. pace of delivery), and speech (e.g. style and content). These changes and interactions, often in combination, humanised lecturers for students. Lecturers’ absence alerted some students to their lecturers’ wellbeing: “it sort of only comes really into view when, like, if they’ve been absent for a while” (P6).

Changes in lecturer behaviour, demeanour, mood, or energy levels made some students cognizant of their lecturers’ wellbeing. One student was alerted by behaviour “actively demonstrating that their wellbeing is maybe not the greatest at that time, so (…) coming in very frazzled, and then the lecture being a little bit all over the place” (P15). Another student recounted:

*A lecturer that usually has a lot of energy and is like very, very passionate about what they're teaching. And they’ll come to class and they’ll be very low energy. And they’ll just seem like very tired and not because they are sick or something (…) they just seem like, just down and [you] can definitely see [it] in their teaching and [it] changes the atmosphere completely (…) And that’s when you kind of wonder, like, ‘oh, maybe something’s going on? Maybe they’re not having a good day’.* (P7)

One student could “see the humanness in [the lecturer]” when “I think they are actually really tired” and “you can feel like they’re trying (…) their best” (P14). Another student reflected, “They look like zombies when they’re standing there with their like mug of coffee and getting through the lecture. And even though they may be trying to, um, show the enthusiasm, you just see physically they’re tired” (P4).

Many students described heightened awareness of lecturer wellbeing due to industrial action by academics in UK universities^1^ “With the strikes, it does make me think twice about my lecturer’s wellbeing and like their working conditions” (P36). Several students had become aware of their lecturer wellbeing because of what lecturers told them about their circumstances, working conditions, and reasons for striking: “when we were told kind of why they were striking or kind of the concerns they had (…) then you do realise, um, what they mean in terms of their wellbeing” (P10).

A few students had never considered lecturer wellbeing. One student explained, “To me the lecturers are just, (…) they’re like a source of information” (P23). Another student attributed “institutional barriers” and boundaries that they thought important to the student–lecturer relationship as being the reason why lecturer wellbeing had “never come to my notice before”; “because we are to maintain this sort of professional distance from them, which helps with their objectivity as well while assessing our marks” (P21). Similarly, a few other students (e.g., P13, P20, P41) intimated a tension between being aware of and feeling empathy for their lecturers’ wellbeing while retaining a professional relationship.

*There’s sort of like the level of professionality that we have to keep. But (…) we found out that one of their children was going through something (…) and so [the lecturer] couldn't teach a few lectures then that made me realise, oh, (…) they do have a life. (…) oh, I really hope that my lecturer is doing fine*. (P20)

### Theme 2: “It feels like a lot of pressure happening at the same time”—A complex range of factors impact lecturer wellbeing

Across the focus groups, students identified multiple factors they believed impacted on their lecturers’ wellbeing. These factors were clustered into three broad categories: (1) university organisational culture and work patterns, (2) student behaviour, and (3) factors external to the university.

#### University organisational culture and work demands

Several students attributed university working practices as influential on their lecturers’ behaviour, emotions, energy levels, communication, and by extension their lecturers’ good or poor wellbeing. First, workload, reward, and recognition were linked with lecturer wellbeing. Many lecturers were noticeably “super, super stressed with the workload” (P3). Several students felt that the volume and breadth of lecturers’ responsibilities negatively impacted their wellbeing, “it feels like a lot of pressure happening at the same time” (P29). Lecturers were perceived to be juggling, “teaching, preparing lectures, updating them, seminars (…) Responding to emails (…) Then actually doing research which is so important for your progression in your job” (P29).

Second, students suggested that “how [lecturers are] treated by the university” (P35) was problematic for lecturer wellbeing, “having a big workload and not being rewarded enough for that negatively impacts lecturer mental wellbeing” (P9). Poor lecturer wellbeing was also associated with not being “fairly compensated” (P14), “pay and working hours” (P14), “short-term contracts and the job insecurity” (P29), and “casualisation, pensions being slashed” (P35). Some students perceived that lecturers could feel undervalued by university management because they were assigned time-diverting administrative tasks that were not directly related to their teaching and research expertise:

*It feels a little bit like the uni doesn’t want to hire more people. You know, ‘why get some kind of administrative assistant in if you can just make one of the professors do it?’, which feels like kind of a waste of talent and time. (…) if you've got all this other stuff to be doing and kind of research on top of all your teaching, then why should you be (…) accepting [mitigating/extenuating circumstances form] deadlines, which you don’t really need any training for?* (P35)

Some students attributed lecturer poor wellbeing to insufficient staffing (P38) or time to mark within contracted working hours, “they’re putting in a whole day and then going home to put in another day” (P1).

Third, students’ observations suggested that when lecturers lacked control over their teaching, lecturers’ wellbeing was impacted negatively. Examples included miscommunication and administrative errors, timetabling, technology, and class size. For instance, a lecturer exhibiting frustration with “timetabling and the wrong person being placed in their class (…) and the admin team being slow to address that” (P41). One student explained that their lecturers were already juggling back-to-back timetabled-teaching and content-heavy curriculum demanded by a professionally accredited course. These administrative errors exacerbated lecturers’ time-induced pressures:

*And so, it can be stressful for the lecturers as well, because it's not necessarily their fault and (…) that should [be] admin stuff. (…). Maybe things weren't communicated to them, and then they have to sort of make up for the lecture we’ve missed (…) so there's like confusion (. …) we do have, like, a lot of lectures that are sort of like back-to-back and (…) it's not great for those lecturers because they do feel it's a lot to get through*. (P12)

One student whose lectures included several hundred students thought that “class size really matters” for lecturer wellbeing, “I think in a small class the lecturer would feel more connected to students” (P23).

Relatedly, several students believed that the timetable structure contributed to poor lecturer wellbeing, “timetabling is (…) the main one because (…) as soon as they had to finish the lecture, they’ll be having to dash off to another lecture straight after for probably a different module that they're doing. And as the weeks go by (…) you see they’re mentally tired” (P4). One student felt concerned when their lecturer did not “even have a lunchtime” (P3). Another student noted that long teaching days along with the flexibility afforded by online working could impact negatively on lecturer wellbeing by blurring boundaries between the competing responsibilities of the lecturer’s work and personal life:

*Sometimes we write essays at 1:00 AM. That’s fine. But I think if it’s your job and it’s your life and you’ve got kids, a spouse, a family to look after, you’ve got to commute. You don’t really wanna be answering emails all the time. (…) I just think the whole online thing, working late, it just extends working hours, which can’t be good for anyone’s wellbeing*. (P15)

In contrast, a student suggested that course-related social opportunities supported lecturer wellbeing:

*I’ve noticed like having good, meaningful connections and feeling like a part of the community can really, you know, make you feel good and (…) keep your mental wellbeing balanced. And (…) all the lecturers feel like they're big like one big community and they keep having so many events where you know, lecturers and students meet and interact together.* (P9)

#### Student behaviour

Student behaviour was identified as another important factor impacting on lecturer wellbeing, “Students can have a very, very big effect on wellbeing of the lecturers” (P3). Student absenteeism and presenteeism were frequently mentioned as being powerful influences on lecturer wellbeing, often in combination and often having similar effects. The level of student engagement, their preparedness for classes, and responsiveness during class also affected their lecturers’ wellbeing.

Many students attributed lecturer unhappiness to student behaviour. Receiving negative student feedback (P17), “complaining about how they’re delivering it” (P32), or “‘Oh, the content’s not engaging’ or ‘I need more of this or more of that’” (P17) impacted adversely on lecturer wellbeing, “The students can be quite rude to some lecturers (…) it really like made her feel very bad because she puts a lot of time and effort into making sure that we're very happy” (P3). Additionally, some lecturers seemed to feel burdened to promise unrealistic marking deadlines, “lecturers do experience pressure to get things back to us as quick as possible (…) whether it's by the students or by the lecturers themselves” (P40).

Lack of student engagement was “definitely a big factor” (P22) associated with lecturer wellbeing. Students not arriving prepared for class noticeably impacted lecturer mood, “the class didn’t do the readings and we could see that she was very disappointed and almost sad about it” (P2). Lecturers became visibly “sad” because “attendance has gone like downhill” (P19), or “annoyed”, “frustrated” (P22), or “more discouraged” (P5), because of “the amount of interaction (…) from their students” (P5). “Either, like, they don’t show up or if (…) the lecturer asks a question and we’re all just kind of sat there dead (. …) I think that tends to happen quite a lot” (P15). Participants believed that these student behaviours impacted lecturer wellbeing negatively, particularly because the students’ behaviours undermined a constructive and respectful learning atmosphere:

*It’s [the lecturer’s] passion. And a lot of the time they’re teaching students that aren’t really interested in the subject, or maybe don’t seem to be interested, might be a little bit demoralising for [the lecturer].* (P14)

In contrast, student behaviour could positively influence lecturer mood, cognition, and behaviour:

*I think the relationships and especially at the end of lectures when, um, students are engaged in actually asking questions or when the lecturers asking questions to us and want to see if we've engaged when many students are participating, you can really see the lecturer, um, you know is glad and actually likes those interactions.* (P10)

Students seeking support outside the class could also impact lecturers’ wellbeing by compounding their workload. “They have a lot, a huge workload. So if there is any interaction that is like outside of that lecture space, it does sort of impact on their own personal time as well” (P12):

*Some of our lecturers get quite frustrated when we e-mail them personally (…) Because obviously (…) that response only goes to you, whereas if you put it on the forum, it obviously is there for everyone to see.* (P40)

#### External factors.

Third, a few students identified external factors that impact on their lecturers’ wellbeing such as, “health and family issues” (P32) and “caregiving responsibilities” (P6). Some students attributed their lecturers’ good wellbeing to being able to interact with their students in-person compared with being compelled to teach online during the COVID-19 pandemic: “you can see the lecturers actually enjoying talking to us in, in the class and having people respond” (P4).

### Theme 3: Lecturer-student wellbeing and (dis)connection – “It’s a bit of a cycle”

Lecturer wellbeing affected student wellbeing, “it really does impact us, the wellbeing of the lecturer in many, many ways” (P34). Students seemed to associate good student wellbeing with constructive lecturer-student interactions, and participating in a positive atmosphere conducive to learning well and feeling good about learning. Features of lecturer wellbeing, such as mood, cognition, behaviour, and health, were identified as contributors to students’ wellbeing. These factors, separately or in combination, impacted on the character of the lecturer-student interactions and lecturer and student wellbeing through a “domino effect” (P34), “feedback loop” (P31), or “cycle” (P17). The impacts on students’ wellbeing included mood, engagement, interest, and understanding.

Lecturer mood impacted student wellbeing in terms of their mood and intellectual engagement during and after class. One student could “immediately tell the lecturer is happy to be there and the energy that put in. It (…) lights up the room and gets everybody talking and gets all the students engaged” (P1). Students associated their lecturer and their own wellbeing with the enjoyment of learning, “It’s just the engagement. (…) like the satisfaction of (…), it can be learning and fun at the same time” (P1). Similarly, if a lecturer’s “wellbeing was good”, they “radiate[d] positive energy” so that one student was “more inclined to be committed to going to the lectures every week” (P14). Conversely:

*if the lecturer, (…) isn’t in a good wellbeing space (…) then that can really change*

*(…) set the mood for the whole room. And if everyone’s feeling down, well then, that’s how you feel, and then you’re not willing to learn. (P20)*

Good lecturer wellbeing was also associated with teaching and learning, with implications for student wellbeing, “if a lecturer’s wellbeing is better, their teaching will be better” (P28). Another explained:

*When your lecturer is, has a good wellbeing, you can see that in the tone of voice (. …) some lecturers, because they’re so passionate about what they’re talking about, they’ll have a clear structure. (…) they’ll introduce and then they’ll go through every single point”* (P20).

Many students linked student and lecturer wellbeing with lecturers who were approachable *and* available.

*If the lecturer has like a better wellbeing, then they’re more likely to take the time out to reply and like, be helpful to students as well. (…) so, um, the better like the lecturer’s wellbeing the better, like, the student’s wellbeing will be as well.* (P18)

When poor lecturer wellbeing results in class cancellation, students perceived that this type of unavailability impacted adversely on their wellbeing as students, “the most obvious ways, (…) if they’re not happy, they go on strike and we miss out on teaching. That’s the kind of the big one” (P35). Students “worried” that this had long-term effects beyond university, “feeling where you know your knowledge is incomplete and I guess it’s just you don’t feel properly qualified or very good about it” (P11).

Lecturers who were perceived as unapproachable impacted negatively on student wellbeing. Lecturer mood communicated lecturer approachability, “they will say (…) ‘you can come in and chat and get advice about, you know, anything’. (…) if they’re more irritable during their lectures, or if they don’t want to interact with us as much, it sort of stops me from contacting them outside of the lecture” (P5). Other students explained the impact on them, “But then if they’re unapproachable then I might be scared to ask that question, [pause] which will cause even more stress” (P8). By implication, student and lecturer wellbeing seemed intrinsically linked through lecturer approachability.

*I’ve definitely experienced that fear before writing an email (…) feeling like you're gonna be annoying to them. And it really makes you second guess yourself (…) you feel isolated when you’re studying.* (P6)

This student also found “sitting in a lecture where like you can’t ask questions and then like not understanding it (…) so disheartening” (P6).

Students’ enthusiasm for the subject during class could influence lecturer approachability and availability to discuss questions during and after class, further impacting on learning. One student reflected on how poor student engagement could be counter-productive for both student and lecturer wellbeing in a “feedback loop”, involving mood, cognition, behaviour, and lecturer-student interactions.

*If we’re not engaging with them, they just feel like they’re teaching a bunch of robots (…) And if the students engage with them better, I feel like they come out of the lecture happy. Like they’ve done a good job. (…) That was worth it. (…) He was just like, ‘I’ll do a Q&A at the end, but you guys have been so interactive. It’s honestly, it’s been great’.* (P1)

In contrast, students’ behaviour could impact adversely on their lecturers’ wellbeing, such that the lecturer withdrew from the students, which subsequently negatively impacted on other students:

*One time I saw like a real time change in the lecturer’s behaviour. The class started out well, but then he had like, a poll (. …) And there were like a couple of people who weren't serious (…) [the lecturer] was just so distracted (. …) In the first half, he was able to articulate it a lot better, um, in a much calmer tone in a way which helped me understand and process all of that information better. But (…) in the second half, he just tried to speed up things a bit, so it was much more difficult for me to grasp on ideas and stuff that he was talking about.* (P23)

A reciprocal relationship between lecturer and student wellbeing was further indicated by students’ descriptions in several focus groups, termed by one student as “a bit of a cycle”. One student’s illustration of this cyclical process started with lecturers not feeling valued by students, “when the lecturer doesn’t feel maybe as appreciated, it just makes (…) the entire lecture, very like, awkward. You might even try to like avoid it, if you feel like, ‘oh, they’re not gonna be in a good mood today’. Like, ‘I don’t wanna deal with it’” (P17). The same student further explained how their lecturer’s discomfort in student–lecturer interactions could lead to lower student attendance, and so negatively affect lecturer wellbeing:

*If you know that it’s gonna be really awkward in the classroom because no one ever speaks, (…) like you might miss a lecture (…) they feel even worse after that. And yeah, it's a bit of a cycle (. …) it will impact their wellbeing even more.* (P17)

A student in a different focus group also connected student–lecturer behaviour and lecturer-student affect as a negative cycle:

*It’s a feedback loop, right? So, if the students are dragging the mood down, then the lecturer or seminar leader will have trouble continuing, maintaining engagement, and then it’s not a great experience either if the professor’s not, um, in a great mood*. (P31)

Several students explained how lecturer wellbeing influenced by university structures or external factors also impacted student wellbeing. When circumstances such as workload caused lecturers to rush through a lecture, it meant that students did not develop depth of understanding or felt unable to ask questions. One student perceived that their own academic performance was suffering because a lecturer had introduced peer marking to alleviate the lecturer’s own workload-stress (P3). Additionally, when lecturers were given insufficient resources to meet marking deadlines, students could also become “a bit anxious” when feedback was not made available on time, “deadlines can be stressful for them, for the lecturers. But at the same time, it can be quite stressful for students as well if they're not been given enough warning” (P41). Several students also commented on how they found themselves absorbing their lecturers’ mood and energy levels. A lecturer who was scheduled to teach late in the day, **“**keeps repeating that she’s not really happy to be there and (…) that she’s tired. And then I (…) started feeling the same way as she’s feeling. It’s almost like her state is kind of like rubbing onto us” (P7). When another student’s lecturer was unhappy their mood was “hostile” and their “class discussions kind of feel less open, kind of less friendly” (P35).

Whatever the cause, several students described how they modified their own behaviour during and after class to minimise any impact on their lecturer.

*I think if the lecturer’s more open and if you can see that they’re visibly in a better mood, you’re more likely to ask a question if something confuses you and interact. Because if they’re not in such a good mood, you don’t really want to disturb them, or make them feel worse, or they might be tired* (P34)

Not all students perceived accommodating for lecturer wellbeing as necessarily affecting their own learning and wellbeing negatively. A few students explained how awareness of their lecturer’s workload compelled them to think more carefully about asking their lecturers’ questions: “I think about, is it actually worth asking just because I know that they already have like such a high workload” (P30). One student thought that this “helps me create more complicated questions” (P27). Another student who felt embarrassed for lecturers when students were not participating in class would “find myself contributing more (…) And I guess that does in turn benefit me because then I’m getting more out of the session by forcing myself to contribute where, whereas I wouldn’t usually want to speak up” (P26).

Students’ empathy for their lecturers’ wellbeing sometimes appeared to impact some students’ learning and wellbeing, “I end up feeling a little bit like we as students are kind of a bit of a burden. We’re sort of just another thing that they have to do in the midst of their crazy schedule” (P28). Several students felt empathetic towards lecturers when they were experiencing poor wellbeing, “when professors are going through a hard time, I think students generally also just feel empathetic and try to put in their effort, as much as they can” (P37). When poor lecturer wellbeing manifested itself in the lecturer’s unavailability (e.g. absence) or unapproachability, the students’ wellbeing was impacted: “the lecturer wellbeing on that day of them being, like, stressed does have a bit of a negative impact on, like, the students, because you do like, kind of empathise with them” (P28).

*Obviously, we are human and if someone else is going through something tough, we feel sorry for them and we have empathy. But at the same time and from the student’s perspective, we have deadlines, we have exams and the same system putting pressure on them is putting pressure on us. (. …) So, if there was support for that lecturer then possibly that support would kind of have like a domino effect and kind of help us all down the line.* (P34)

## Discussion

This study explored students’ perceptions of their lecturers’ wellbeing and how this may relate to student wellbeing. Students understand their lecturers’ wellbeing, that is, whether their lecturers are “feeling good and functioning well” (Ruggeri et al., 2020), from what lecturers do, what lecturers say, and how they say it. Lecturer-student interactions enabled students to see lecturers as human, to identify a complex range of factors impacting lecturer wellbeing, and to notice the interdependence of lecturer wellbeing and student wellbeing. Students seemed to think that this type of “human connection” could benefit their wellbeing, as learners. When students deduced that a lecturer had good wellbeing, students perceived lecturers as being more approachable for constructive interactions that benefited their learning and wellbeing.

Our focus groups aligned with previous findings that teaching and learning are relational processes (Eloff et al., 2021; Hagenauer et al., 2023) and that the quality of the lecturer-student relationship is important for university students’ wellbeing (Trolian et al., 2020) whilst elucidating students’ perspectives on which aspects of lecturer and student wellbeing contribute to the relationship. Namely, affective, cognitive, and behavioural aspects of lecturer wellbeing impacted the quality of the relationship between lecturers and students.

Our participants reflected on their relationships with lecturers at length, repeatedly circling back to aspects of their relationship with lectures to both think about how they understood their lecturers’ wellbeing and the impact this had on them. These findings align with studies about teacher-student relationships, in which reciprocal emotion transmission between teachers and students was interpreted from the others’ behaviour (Frenzel et al., 2018). That is, it is through the relationship between teachers and their students that the wellbeing of one individual may influence the wellbeing of another. For example, some participants described a cyclical process of students becoming more engaged and motivated after noticing their lecturers’ positive emotion through their enthusiasm and passion for the subject. In turn, their lecturers’ mood and behaviour became more positive in response the students’ engagement. Students perceived that when lecturers had good wellbeing, lecturers taught more effectively. When classroom mood was positive, students and lecturers explored the subject in greater depth and from different perspectives. This included being encouraged to think critically. Research at the intersection between affect and cognition has demonstrated that that mood states can change attention and intentions to act (Fredrickson & Branigan, 2005). Conversely, many students noticed the reciprocal effect of poor student engagement on lecturer wellbeing, whereby some lecturers displayed negative emotions and became less approachable. This interplay between lecturer and student mood, cognition and behaviour further highlight that student learning is relational and can involve the transmission of different emotions (Pekrun & Linnenbrink-Garcia, 2012).

In the context of the neo-liberal managerial university that focuses on individual attainment and satisfaction (Graham & Moir, 2022), it might be worth remembering the power of relational pedagogy, which recognises meaningful relationships as fundamental to effective learning and teaching (Bovill, 2020; Gravett et al., 2021). Several students identified job demands such as high lecturer workload and student behaviour as detrimental to their lecturers’ wellbeing, chiming with findings from studies with academics (Johnson et al., 2019; Urbina-Garcia, 2020). Some students seemed to associate demanding lecturer workload with low quality lecturer-student interactions and in turn both poor lecturer and student wellbeing. For instance, some students linked lecturer workload to lecturer behaviour such as arriving late to teach, hurrying through core curricula, and having insufficient time to answer or ask questions to check for student understanding, or delve deeper into the subject-matter. Under these conditions, students often felt the learning atmosphere was more negative and that lecturers became less approachable. This could reflect lecturers’ emotional exhaustion or depersonalisation of students, which can be indicators of lecturer burnout (Bakker et al., 2023; Watts & Robertson, 2011). Interview studies with lecturers suggest that lecturer approachability is important for developing positive teacher-student relationships and provides students with academic support (Hagenauer et al., 2023). Likewise, qualitative research with students reports that negative experiences of lecturer-student relationships often involve unresponsiveness from lecturers (Snijders et al., 2022).

Interactions built student empathy for lecturers. Some students’ expressions of empathy were striking. Several students were concerned about the impact that their behaviour could have on lecturer wellbeing (Theme 2), and so sometimes changed their own behaviour (Theme 3). The role of teacher empathy for student cognitive and psychosocial outcomes has been researched (Aldrup et al., 2022). However, our study suggests that the role of student empathy for lecturers and students is worth further examination.

Consistent with research with lecturers, our student participants perceived that a range of organisational practices and external factors can impact lecturer wellbeing (Hammoudi Halat et al., 2023; Urbina-Garcia, 2020). Class size, learning in-person, timetabling, and lecturer reward and recognition seemed to contribute to students’ experience, insight, and reactions to their lecturers. These patterns are consistent with research showing that university policies and procedures that prioritise large class sizes have high staff-student ratios and an unrealistic regime of teaching, marking, administration, and research impact lecturer wellbeing negatively (Hammoudi Halat et al., 2023; Kinman & Wray, 2021). Insights from our focus groups indicate that students want to be taught by lecturers who are supported and valued by their organisation and students.

### Limitations and future research

In light of our research question, lecturer wellbeing was explored only from the student perspective. It was interesting that several students showed awareness of some potential influences on lecturer wellbeing that align with evidence from studies with lecturers (Fontinha et al., 2019; Hammoudi Halat et al., 2023; Johnson et al., 2019; Urbina-Garcia, 2020). Nonetheless, students are unlikely to have a complete understanding of factors contributing to lecturer wellbeing, including lecturers’ individual differences such as lecturer perfectionism and coping strategies such as job crafting (Bakker et al., 2023).

The sample represented a diverse range of ethnicities, subject areas, and year-groups, thereby facilitating a range of perspectives on the subject. However, it is likely that only those interested in the topic opted to participate. Moreover, most participants were female and all came from two research-intensive universities within the UK. Consequently, participants from other universities may have revealed different themes.

Because ‘wellbeing’ is a broad and multifaceted construct understood in different ways by different individuals, future studies could explore the consequences of the lecturer-student relationship both qualitatively and quantitatively using a uniform definition of wellbeing with participants. For instance, it may be helpful to support participants to differentiate between facets of wellbeing such as hedonic wellbeing and eudaimonic wellbeing (Ruggeri et al., 2020).

To address all these limitations above, different methods and populations could be used to examine the prevalence of the experiences that the students described and the cause-effect relationships impacting *both* lecturer and student wellbeing. For example, testing the cyclical relationship between teacher and student wellbeing may require a large-scale longitudinal study, tracking teacher, and student wellbeing over an academic year.

## Implications and conclusion

This study contributes to understanding of what adopting “a whole university approach” means in practice. Because lecturer and student wellbeing are interdependent, it is likely that if universities help lecturer wellbeing to improve, this will benefit student wellbeing, including deeper learning. As policy discussions in the higher education sector continue to consider how institutions can better support students, connecting with their learners more compassionately (Higher Education Student Support Champion, 2023), our data suggests that considering lecturer wellbeing and lecturer-student interactions is paramount.

Our data suggest that investigating the effectiveness of strategies to enable positive student–lecturer interactions may promote both student and lecturer wellbeing. To this end, insights from our student focus groups suggest four possible ways to support lecturer wellbeing and student wellbeing simultaneously, as part of a whole university approach. First, provide lecturers with sufficient time to engage constructively with their students and alongside their other contractual responsibilities such as research. This could include considering timetabling, deadlines for marking, investing in more efficient systems, or recruiting more administrative and/or teaching staff. Second, protect student-staff ratios and schedule smaller class sizes that are conducive to more personalised lecturer-student interactions. Third, work with lecturers and students to identify administrative tasks that could be removed from lecturer workload without compromising student wellbeing. This may include establishing alternative systems for granting student extension requests. Fourth, create policies and practices that support mutually respectful classroom interaction—in which questions can be raised and discussed for deeper insight and understanding. Depending on the requirements of academic disciplines, this could involve students interacting meaningfully with a subject expert consistently throughout each module. Our findings suggest that these four steps could support lecturer approachability and availability and constructive and respectful lecturer-student interactions. Overall, our study indicates that addressing organisational practices is critical for improving staff wellbeing, thereby also enhancing student wellbeing.

## Supplementary Information

Below is the link to the electronic supplementary material.

Supplementary file1 (DOCX 43 KB)

## Data Availability

The topic guide is included in the Supplementary Materials. Transcripts will be made available via OSF.

## Footnotes

1 Lecturers part of the UK’s University and College Union were engaged in industrial action throughout academic year 2022/2023, commencing September 2022; https://www.ucu.org.uk.
