# Supplementary material for: Their wellbeing affects our wellbeing: student perspectives of lecturer wellbeing and its consequences for student wellbeing
Source: High Educ (Dordr). 2024 Dec 14;90(4):1065–82. doi: 10.1007/s10734-024-01365-0 (PMC12592275; doi:10.1007/s10734-024-01365-0)
Supplement: Supplementary file 1 — Supplementary file1 (DOCX 43 KB) [file 10734_2024_1365_MOESM1_ESM.docx]

**Supplementary Materials.**

**Manuscript title:** “Their wellbeing affects our wellbeing: Student perspectives of lecturer wellbeing and its consequences for student wellbeing”

**Journal:** Higher Education

**Table S1: Participant subject area based on Higher Education Statistics Agency (HESA) categories^a^ (N=41)**

| **HESA group allocation Level 1** | **Frequency** |
| --- | --- |
| Subjects allied to medicine | 7 |
| Medicine and dentistry | 6 |
| Social sciences | 5 |
| Geography, earth and environmental studies (natural sciences & social sciences) | 5 |
| Law | 4 |
| Engineering and Technology | 2 |
| Business and management | 2 |
| Historical, philosophical and religious studies | 2 |
| Biological and Sports Sciences | 1 |
| Psychology | 1 |
| Finance | 1 |
| Physical sciences | 1 |
| Computing | 1 |
| Literature in English | 1 |
| Media, Journalism and communications | 1 |
| Design, and creative and performing arts | 1 |

^a^Participants’ self-reported subject areas were allocated to the HESA categories by Authors 1 & 2.

**Table S2: Participants’ ethnicity (N=41)**

| **Ethnicity** | **N** |
| --- | --- |
| Asian or Asian British | 22 |
| White or Caucasian | 11 |
| Mixed | 2 |
| Black or Black British | 1 |
| Chinese or Chinese British | 1 |
| None of the above. I describe my ethnic origin as... (please specify below.) | 4 |
| Arab^a^ | 1 |
| Middle eastern / White^a^ | 1 |
| North African^a^ | 1 |
| Not specified^a^ | 1 |

^a^Categories added by participants

**Table S3: Disabilities declared (participants could select more than one) (N=41)**

| **Declared disability** | **Frequency** |
| --- | --- |
| None of the above apply to me. | 28 |
| I have a physical disability or illness (e.g., asthma) | 4 |
| I have a long-term mental health condition (e.g., depression or anxiety) | 3 |
| I have a learning difference such as dyslexia or dyspraxia | 2 |
| I have a sensory disability or illness (e.g., hearing impairment) | 1 |
| I have another long-term condition, diagnosis or illness. (Please specify below.) |  |
| ADHD^a^ | 1 |
| Back condition^a^ | 1 |
| Prefer not to say | 1 |
| No response provided | 1 |

^a^Categories added by participants

**S4: Focus Group Topic Guide (indicative)**

Question 1 - **To what extent do you consider lecturer wellbeing when you interact with your lecturers?**

Prompts - Do you think about it a lot? Not thought about it before? Please tell me why this is interesting or important to you? (Possible prompts: impact on learning, academic performance, engagement with studies, feeling part of an academic community, feelings that they can get the support they need for studies or wellbeing?)

Question 2 - Given our discussion**, what factors that you think impact on lecturer wellbeing, positively or negatively’?** Which factors are particularly important? Please explain reason.

Question 3 - **Do you think lecturer wellbeing impacts on your experiences as a student?**

(NB – to protect participant MH and MWB, don’t ask for specific examples.)

Possible prompts: In your experience as a student, has lecturer wellbeing impacted on your

- - Your learning?
  - Your academic performance
  - Your engagement with your studies?
  - Your feeling part of an academic community?
  - feelings that you can get the support you need as a student?

Question 4 - We have come to the end of the session. Is there anything you would like to add?

Question 5 - Thank you for participating – and reminder about online voucher.

**S5: Additional evidence for themes.**

| **Theme 1: “Wait, these are actually people that are working” - Human connection** | |
| --- | --- |
| Noticing lecturer wellbeing online and in-person – because of lecturer difficulty in communicating, personal experience | P4: Even though I didn't have that relationship because I've never seen them in-person, they were still these lecturers and and we did a lot of Q&A's in person as well. And you can see from like the way that they're talking and the struggles that they're having to communicate. And I'd be like, “OK, maybe, Is like is everything OK?”. |
| Notice lecturer wellbeing b/c change mood or way teach – more irritable or softly spoken | P7: I've noticed that some lecturers become a lot more irritable when they're like, not as in a good mood. Like if there's usually if there's people that are talking, they wouldn't, they wouldn't bother them. But then when you can tell that they're not having a great day, they'll like start pointing out every single person that's like not listening to them. And there's like other lecturers that are not as, that won't verbalize it as much where it's just like, like I said before like they'll just like kind of speak more quiet[ly]. |
| Noticing lecturer wellbeing- b/c way arrived class and organised | P31: the lecturers seem to have a lot on their plate (…) if they seem, if ordinarily they are, um, quite organised but they seem, you know, at a certain period of time to have, um, a lot to do or they seem to be, err, or they always seem to have very little time, for example, coming a little late and leaving early, or if they emotionally seem stressed. |
| Noticing wellbeing – depends on personal connection, interactions, knowing them | P12: I haven't really thought about lecturer wellbeing, aside from kind of like during COVID. (…) I would say this year, (…) But I would say on like cos we have placements this year. So (…) it's kind of, um, like the people who teach us on placements and who supervise us and stuff, I do tend to think about them a lot, because like if they're unwell or if they seem stressed and stuff, because we have a much more personal relationship**.** But with lecturers, you don't really interact like that. |
| Noticing lecturer wellbeing b/c of strikes | P35: I think it's really come to the fore with the UCU strikes in particular, (…) So I think in particular that's kind of made us all very kind of acutely aware of, yeah, their wellbeing.  P14: “They do talk about the strikes a lot and like the unionisation of like lecturers and teachers.”  P29: “I’ve thought about [lecturer wellbeing] quite a lot this year with the strikes because some lecturers have been quite open to us about the fact that they actually would like to strike”  P22: “they've given sort of short presentations on why they're striking and having to do, you know, so many hours, which are they're not really compensated for properly”.  P9: “There's been so many strikes this year (…) and more information on why they are striking, and I think, because of that, I've been thinking more about lecturer wellbeing” |
| Notice wellbeing - only when classes cancelled | P33: I don’t really get the chance to personally connect with lecturers, so I haven’t really given a lot of thought. But yeah, sometimes we generally do have our classes cancelled, just on short notice and that is when I think about it, otherwise I don’t. |
| Noticing lecturer wellbeing b/c of lecturer response to student attendance | P18: I think, I don't think it's something I think about like a lot, but I think you know, sometimes like especially after COVID, when like the attendance to lectures and stuff like that has gone down. I've thought about it then. |
|  | P19: I didn't attend one of my lectures and then after I had like a meeting because the lecturer was also my personal tutor. And they just said like they wanted like me to e-mail them just to say I was OK. So I didn't think about the lecturer worrying about me not attending their lesson or like, thinking anything of it. |
| Noticing lecturer wellbeing b/c of personal circumstance | P24: Also when considering when to send emails and stuff, trying to be respectful. Also, we had one who was pregnant. |
| Noticing lecturer wellbeing, hesitant to ask after lecturer wellbeing, tension between formal yet empathetic relationship | P13: it's just I guess it's like it feels kind of awkward to just like ask them how they're doing and stuff like that. Cos I like so I guess we don't know if that's like appropriate or not. |

| **Theme 2:** **“It feels like a lot of pressure happening at the same time” - A complex range of factors impact lecturer wellbeing** | |
| --- | --- |
| 2.1 University organisational culture and working practices | |
| Workload, inc. curriculum | P12: because we have quite a lot of content to get through and I think they're sort of like a pressure on the lecturers to like get through this as quickly as possible. Even though sometimes they do express to us that they, like, might not agree with us learning this much, complaining and stuff like. So there can be like sort of a rush to kind of get through a lot of the stuff. Um I think lecturers sometimes they just like get through a bunch of stuff, where they’re like, “well, I'm running out time so, like, you can read this in your own time”. And I know that's not necessarily their fault. It's just like, so maybe there’s like an unrealistic amount, um, is expected of them. |
| Admin – timetabling | P1: I would say there was one of the professors I had and he was quite upset about the timetabling. (…) But the professor came back to me saying yeah, the other professor’s been really upset because of the timetabling and the room changes and all of that was in first semester. |
| Marking deadlines | P38: I feel like that pressure is also increased when (…) sometimes they e-mail us and they're like, “oh, we'll get it back by the end of February”, (…) And then we pass that like self-assigned deadline, and then more people e-mail them asking like where it is. And I think that just piles onto the pressure and makes them feel like they have to get it out as soon as possible. But, obviously it is a lot of work if they're marking it by themselves, which was the case for one of our lecturers. [They] ended up not having enough [postgraduate teaching assistant] to help [them] with the marking. |
| Pay and conditions / tensions associated with striking | P24: with the strikes and everything, I think that was like quite a big thing. I'm talking about how like their pensions and stuff. And a lot of lecturers, my striking lecturers, were saying how they didn't want to strike and like the money they would lose out on and how they didn't enjoy not teaching. So that obviously, I imagine has an impact on their wellbeing. |
| Social events – knowing the environment, colleagues and students - positive emotion | P1: I was going to say having like social events for lecturers, like “meet and greets”. (…) Like that's just for staff to get to know their students. Like at the end of the day, I feel like if they know their environment, they're comfortable in their own environment, they know their students on a personal level, they'll feel a bit more happy coming into work.  P14: “their working environment (…) like how their relationship with like their colleagues and the students are” |
| Timetable structure impact student behaviour, impact lecturer wellbeing | P38: So like time of day obviously affects our attendance as well. So let's say like for example in the middle of the day you have more people attending a session. You see that the lecturers are more like upbeat and they're more like engaging with the with the students themselves compared to like a later session where not many people turn up. |

| 2.2 Student behaviour | |
| --- | --- |
| Student behaviour – not preparing | P19: But sometimes if we haven't done the reading, you can tell the lecturer isn't like, not enthusiastic and not like, [pause] like kind of upset and disappointed that our group isn't talking as much because we don't have like the background information. And then we’re just like looking at the text on our laptops. |
| Student behaviour – impacts lecturer wellbeing. | P22: I think I would agree that student engagement is like definitely a big factor because (…) with my lecturers, like there are some who whenever anyone is sort of talking even quietly, they'll point to try and get quite frustrated (…) But I think because it happens so often and they feel that, yeah, they're not being listened to, so “what's the point in us coming if we're not going to be giving them our full attention?” and times where they’ll ask a question and no one is really like willing to answer it. And you can just see that they're they're kind of getting annoyed that that people aren't like participating properly. |
| Student Behaviour – not valuing lecturer effort | P18: Yeah, I feel like, um, I feel like if there's not that many people who came to the lecture, then it might make the lecturer feel a bit like unappreciated or like they put in so much effort for nothing.  P18: normally like students, they just don’t show up because they just can't be bothered to go in. It's not, It's not, like it's not even like a deeper reason. But I remember like one of the lecturers emailed us saying, like, “Is there something I'm doing wrong?”. Like, “why don't people showing up?”, So (…) I feel like, because we have so many lecturers, we don't think about like one lecturer specifically. We just, like for us it's very easy to just miss a lecture. For the lecturer, it's just like we, we're his class sort of thing. So, it makes more of a, more of an effect on him, if we didn't show up. |

| **Theme 3: Lecturer-student wellbeing and (dis) connection - “It’s a bit of a cycle”** | |
| --- | --- |
| Lecturer wellbeing impacting student wellbeing - learning, focus, motivation | P20: And (…) when a lecturer is in a good mood and they seem motivated and passionate about what they're talking about, it makes me want to listen and want to study and makes me want to interact and reply to questions.  P20: If they're not feeling the best (…) they might skip a few topics and then forget about it and then send you an e-mail over (…) the overall structure, the overall way method of teaching can be disrupted (. …) it kind of disrupts learning.  P32: when [lecturers] are in a positive mood and the atmosphere is a lot nicer, you are more engaged and that would impact your studies (…) if you are in the moment and you’re enjoying the atmosphere, if you’re connecting with person who is teaching and they are making it interactive and enjoyable, then you’re likely to go home and think “oh, that’s interesting” (…) And I’m more likely to focus on the topic |
| Domino effect – student behaviour impacts lecturer approachability – impacts student wellbeing | P21: Sure. So I think when lecturers aren't really feeling well, or when they're not really able to establish a rapport with the class, that's when they stop taking questions outside of class. So (…) during the 10-minute break, some lecturers, when they see that the class is really engaged and really interested in the topic that they're teaching and asking a lot of questions during class listening, listening attentively, that's when they even utilize that break to answer students questions. Um, but (…) because people were not really paying attention or not a lot of people turned up to the lecture, lecturers during that break, they told students that um, “It's my break (…) write an e-mail or consult with your tutor leader” (…) It impacts us in the sense that it hinders our engagement with the course, with the curriculum. (…) well, it just feels rude. (…) we have these burning questions (…) we are clearly motivated to know more about that topic and when the person who is supposed to be our source of introduction and information for that topic isn't, isn't seeing and acknowledging that passion, that curiosity (…) that's a little disheartening. So emotionally you feel a little less involved. |
| Domino effect – lecturer wellbeing [signalled by mood/responsiveness] impacts approachability – students | P8: when I can’t ask the question, I know that when I get home I will have to spend more time on that. And then I'll be worried that it wouldn't be the right answer. So it will cause stress. Um, I have, we do have a Q&A function, but then sometimes when I have asked a question I get a reply that “you should watch the lecture again because the answer was in the lecture” when it is not. So I feel like if I were to ask the question in real life I could get an answer. But then if they're unapproachable then I might be scared to ask that question, [pause] which will cause even more stress. |
|  | P11: I think [lecturer wellbeing] definitely impacts the way probably how responsive they are. There may be questions that I have or how willing they might be to maybe go the extra mile or (…) just respond to any concerns that I might have. |
|  | P16: I don't know if this would particularly fall under wellbeing, but sometimes they would have a cold attitude as if they just want to get over with, if I have, if I'm there with the question. (…) There is this particular lecturer who would try to ask questions, right, and go around to the group of students and ask them individually what their thoughts are. And let's say a student answered (…) He would literally just move on to the next person, like, no emotion, no, like without saying anything, would just move on as if he ignores you. And then, yeah, so the students feel dismissed and then they just don't feel like answering any questions again (…) But at university, there, like the content we are learning is much more complex and we can't just go to our friends. (…) Not being able to ask them questions, it's just very demoralising and it makes you think that if you don't understand something, then just give up. There's no other option or no alternative. |
|  | P24: (…) some of my favourite lecturers would always stop and chat with us if he saw us, like walking down the corridor (…) And he would kind of talk to us about his research, different modules he taught (…) When you have more of a human connection with someone, especially when they're in a position of like authority or power, you just like you get more of the mutual respect. And I think it's easier to kind of learn and engage better when you kind of like someone as a person, even if they are your lecturer. |
| Feedback loop - Tension – approachable, relationship, workload, insufficient time, to help | P12: if you have like good relationships with certain lecturers and some lecturers are very open with getting questions and, um, you can sort of approach them. But then on the flip side, that puts a lot of pressure on them as well because they already have this huge workload to get through. And they also have a tonne of people, um, bombarding them with questions afterwards in the same lectures that they spent ages to [prepare]. |
| Feedback loop – lecturer wellbeing (equated with energy/tiredness) impacts student wellbeing (commitment and enjoyment) | P14: if the lecturer has can like radiate like positive energy and like, so if their wellbeing is good and they kind of give that off in like lectures, I'm more inclined to be committed to going to the lectures every week. (…) whereas with some lecturers, they just like, you can tell that they are really tired or they don't really wanna be there. Um, so then I think, like, I might as well just watch the recording in my own time. I don't need to. I don't feel the need to, like, get up, go to class and everything. So it definitely impacts my commitment and also my enjoyment of the course. |
| Students  modifying behaviour to minimise impact on lecturer wellbeing | P9: I’ve been thinking more about lecturer wellbeing and yeah, I mean it definitely, it's very important. (…) So the way I ask questions or if I ever have to give feedback about our module, I take into consideration that they have put in a lot of effort in organizing it. So yeah, I mean I try to phrase it in a way that's not too “out there” and “up front”. |
|  | P18: So there was one time when, like the person running the teaching, like emailed us back saying that they were quite upset that, like, um, everyone didn't come and everything. So it just makes you think that like, you know, cause for them, especially if you've travelled in and you have put in a lot of effort to, like, make content, make slides and stuff like that and then your students don’t even show up. So, in like, from the student perspective, I, it's made me a lot more conscious about like attending stuff, cos it's like, it's, from my side, It's kind of inconsiderate to just like not show up. |
|  | P26: when I'm when I'm emailing I do (…) think about like the kind of effect that what I'm saying is having on the lecturers. |
|  | P28: We're a cohort of 450 people and I think [that] whenever we have a lecture and there's maybe 20 of us that turn up, I feel bad for the lecturer, and I can imagine that's quite demotivating for them. And I do find myself (…) thinking (…) it's not [a] very nice position for them to be in. And I also find that when I interact with them and ask them questions, I do it so that I can still engage with them because I know it's horrid when you're asking questions and no one ever engages, I think that's, yeah, really demotivating. |
